# Effect of Ozempic on Diabetic Nephropathy: A Case Report

**DOI:** 10.7759/cureus.87416

**Published:** 2025-07-07

**Authors:** Jimmy Joseph

**Affiliations:** 1 Internal Medicine, Aster DM Healthcare, Dubai, ARE

**Keywords:** albuminuria, diabetic nephropathy, estimated glomerular filtration rate (egfr), glp-1 receptor agonist, semaglutide

## Abstract

Semaglutide (Ozempic), a Glucagon-like peptide-1 (GLP-1) receptor agonist, has shown promise in improving glycemic control and offering renal protection in type 2 diabetes. We present the case of a 40-year-old male with poorly controlled type 2 diabetes and early diabetic nephropathy, who experienced significant metabolic and renal improvements following semaglutide therapy. Initially, on SGLT2 inhibitors, the patient discontinued treatment due to recurrent urinary tract infections. On presentation, he exhibited hyperglycemia, dyslipidemia, proteinuria, and reduced eGFR. Semaglutide was initiated alongside metformin, gliclazide, antihypertensives, and lipid-lowering agents. Over 12 months, HbA1c improved from 9.8% to 6.1%, and urine albumin-to-creatinine ratio decreased from 267 mg/g to 34 mg/g, with improved eGFR. This case supports the renoprotective and metabolic benefits of semaglutide and highlights its potential as a therapeutic option in patients intolerant to SGLT2 inhibitors.

## Introduction

Diabetic nephropathy is a leading microvascular complication of type 2 diabetes mellitus (T2DM), contributing significantly to the global burden of end-stage renal disease (ESRD). Its pathogenesis involves persistent hyperglycemia, insulin resistance, systemic inflammation, and oxidative stress, leading to progressive renal damage. While early intervention with glucose-lowering therapies can delay disease progression, few treatments have historically demonstrated direct renoprotective effects. The introduction of sodium-glucose cotransporter-2 (SGLT2) inhibitors marked a paradigm shift in managing diabetic nephropathy due to their proven ability to slow renal function decline and reduce albuminuria [1]. However, their use can be limited by adverse effects such as genital infections and volume depletion, especially in vulnerable populations [2]. Glucagon-like peptide-1 receptor agonists (GLP-1 RAs), such as semaglutide (Ozempic), have emerged as effective alternatives offering not only glycemic and weight reduction benefits but also cardiovascular and potential renal protection [3]. Mechanistically, GLP-1 RAs reduce systemic inflammation, oxidative stress, and improve endothelial function - all key factors implicated in the progression of diabetic nephropathy [4,5]. They also promote natriuresis and have been shown to reduce albuminuria independent of their glucose-lowering effects [6]. Large-scale trials support their efficacy. The SUSTAIN-6 trial showed that semaglutide reduced new or worsening nephropathy by 36% in patients with T2DM [3]. Similarly, the REWIND trial demonstrated a 15% reduction in composite renal outcomes with dulaglutide [7]. Most recently, the FLOW trial provided robust evidence of semaglutide slowing estimated glomerular filtration rate (eGFR) decline and reducing progression to ESRD in patients with diabetic chronic kidney disease (CKD) [8]. Although GLP-1 RAs are not yet first-line therapies for nephropathy, current the American Diabetes Association (ADA) and Kidney Disease: Improving Global Outcomes (KDIGO) guidelines recommend them in patients with T2DM and CKD when SGLT2 inhibitors are contraindicated or insufficient [9,10]. This report presents a case of a middle-aged male with T2DM who was unable to tolerate SGLT2 inhibitors due to recurrent urinary tract infections and subsequently developed early nephropathy. Initiation of semaglutide led to marked improvements in glycemic control, lipid profile, and renal parameters over 12 months, highlighting the valuable role of GLP-1 RAs in complex diabetic patients.

## Case presentation

A 40-year-old Indian male, with a known case of type 2 diabetes mellitus (T2DM) for 3 years, presented with fatigue, polydipsia, and frothy urine for 1.5 months. He reported a weight gain of 8 kg over the past year, irregular diet, ceased exercise, and smoked five cigarettes per day. Past medication history included SGLT2 inhibitors, which were discontinued due to recurrent urinary tract infections (UTIs), and he defaulted from follow-up.

On clinical examination, the patient was morbidly obese with a weight of 132 kg and a body mass index (BMI) of 43 kg/m². His blood pressure was elevated at 156/96 mmHg, consistent with poorly controlled hypertension. Pitting pedal edema was noted bilaterally, suggestive of early fluid overload and possible renal dysfunction. The skin appeared dry, but there were no visible signs of infection. Retina examination revealed no evidence of diabetic retinopathy on initial assessment.

Table 1 summarizes the patient's baseline laboratory findings, indicating poorly controlled diabetes and significant metabolic derangements. The HbA1c of 9.8% and fasting blood sugar of 186 mg/dL reflect chronic hyperglycemia. Renal parameters are deranged, with elevated creatinine (1.8 mg/dL), reduced eGFR (56 mL/min), and markedly increased urine albumin-to-creatinine ratio (ACR) (267 mg/g) and microalbumin (256 mg/L), suggesting early diabetic nephropathy. Lipid profile is markedly abnormal, with elevated total cholesterol, low-density lipoprotein (LDL), and triglycerides, and high-density lipoprotein (HDL), indicating high cardiovascular risk. Liver enzymes (aspartate aminotransferase (AST)/alanine transaminase (ALT)) are significantly raised, suggestive of hepatic involvement, possibly metabolic dysfunction-associated steatohepatitis(MASH)/nonalcoholic fatty liver disease (NAFLD) or steatohepatitis.

**Table 1:** Baseline Laboratory Investigations Hba1c: Glycosylated Hemoglobin; FBS: Fasting blood sugar; GFR: Estimated glomerular filtration rate; ACR Albumin Creatinine ratio; LDL Low-density lipoprotein; HDL: High-density lipoprotein; AST Aspartate transaminase; ALT Alanine transaminase

Parameter	Result	Normal Range
HbA1c	9.8%	<5.7%
FBS	186 mg/dL	70–100 mg/dL
Creatinine	1.8 mg/dL	0.6–1.3 mg/dL
eGFR	56 mL/min	>90 mL/min
Urine ACR	267 mg/g	<30 mg/g
Microalbumin	256 mg/L	<30 mg/L
Total cholesterol	275 mg/dL	<200 mg/dL
LDL	202 mg/dL	<100 mg/dL
HDL	30 mg/dL	>40 mg/dL
Triglycerides	295 mg/dL	<150 mg/dL
AST	98U/L	<40 U/L
ALT	124U/L	<40 U/L

**Figure 1:**
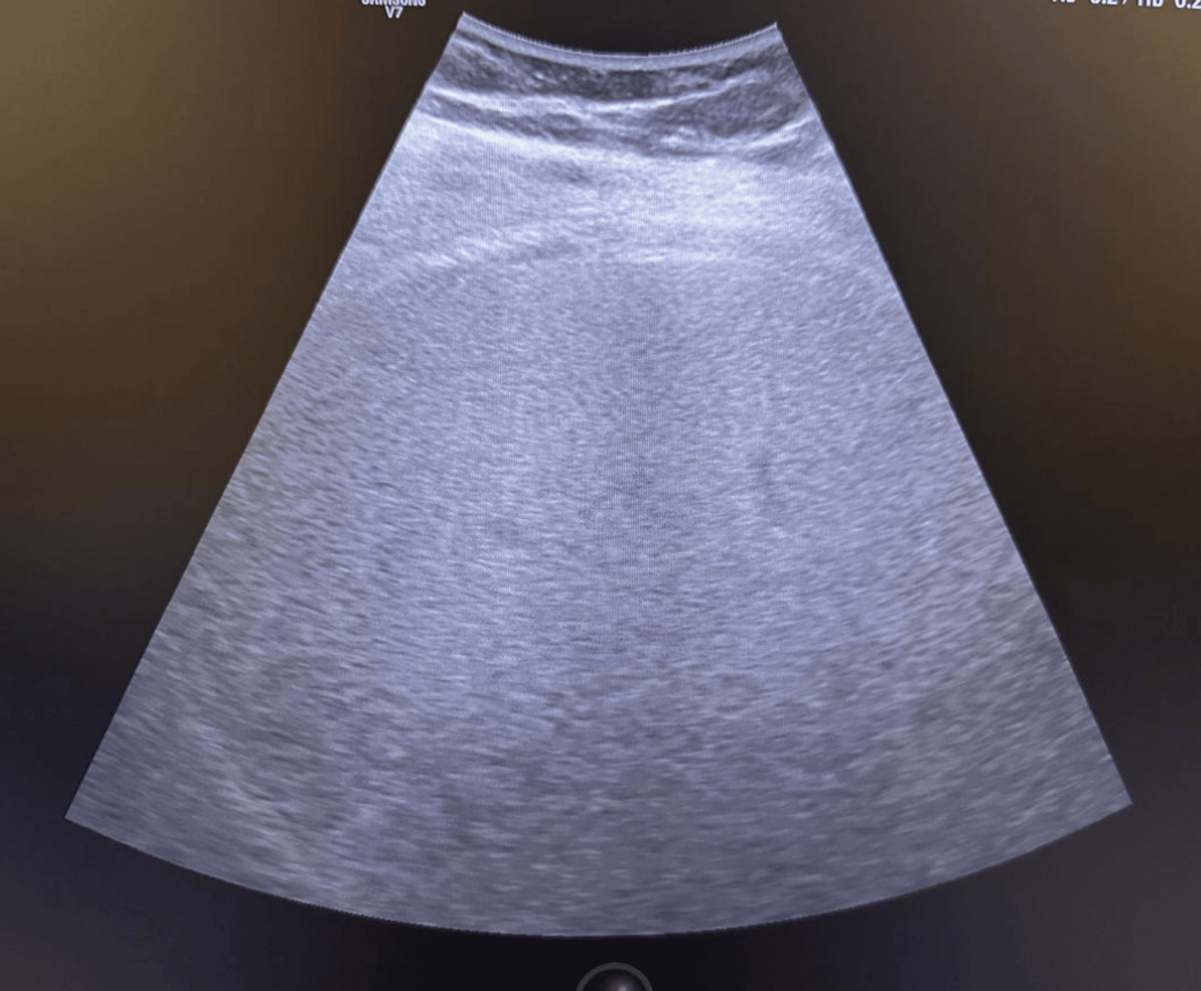
Ultrasound showing grade 2 fatty liver

The ultrasound showed grade 2 fatty liver (Figure 1).

**Figure 2:**
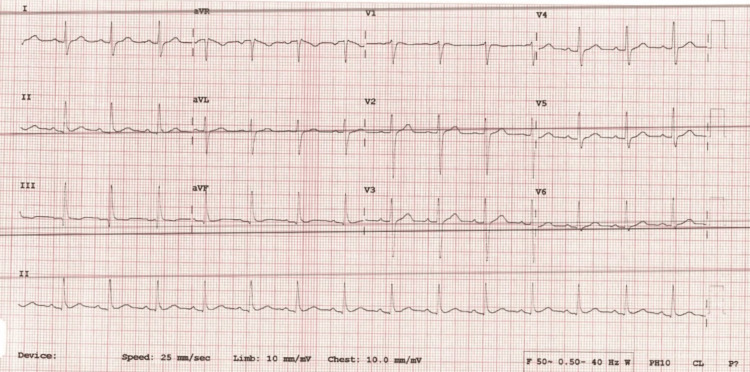
ECG showing sinus rhythm ECG: Electrocardiogram

The ECG showed normal sinus rhythm (Figure 2).

The patient was initiated on a comprehensive treatment regimen targeting hyperglycemia, dyslipidemia, and hypertension. Semaglutide (Ozempic) was started at 0.25 mg subcutaneously and gradually titrated to 1 mg once weekly. Metformin XR (extended release) 500 mg twice daily was continued. Gliclazide MR (modified release) 60 mg once daily was included. Valsartan 80 mg once daily was added for blood pressure control and albuminuria. Atorvastatin 20 mg daily was prescribed to address elevated LDL levels and reduce atherosclerotic risk. Fenofibrate 145 mg daily was used to target hypertriglyceridemia.

The patient demonstrated significant clinical and biochemical improvements over a 12-month period (Table 2).

**Table 2:** Follow-Up Results over 12 months M: Months; Hba1c: Glycosylated hemoglobin; GFR: Glomerular filtration rate; ACR: Albumin creatinine ratio; LDL: Low-density lipoprotein; TG: Triglycerides

Time	HbA1c	eGFR	ACR (mg/g)	Microalbumin (mg/L)	LDL	TG	Weight Loss
0 M	9.8	56	267	256	202	295	-
3 M	7.6	62	186	190	165	196	5 kg
6 M	6.7	66	128	111	130	156	9 kg
9 M	6.4	67	76	70	102	132	11 kg
12 M	6.1	71	34	37	80	128	13 kg

HbA1c steadily declined from 9.8% at baseline to 6.1% at 12 months, reflecting improved glycemic control. Renal function showed progressive improvement, with eGFR increasing from 56 to 71 mL/min and a marked reduction in albuminuria-urine ACR decreased from 267 mg/g to 34 mg/g, and microalbumin from 256 mg/L to 37 mg/L-indicating improvement of diabetic nephropathy. Lipid profile improved considerably: LDL dropped from 202 to 80 mg/dL and triglycerides from 295 to 128 mg/dL, suggesting better cardiovascular risk management. Additionally, the patient experienced a total weight loss of 13 kg over the year, contributing to the overall metabolic benefits. These changes reflect the multidimensional impact of GLP-1 receptor agonist therapy on diabetes-related complications.

The patient tolerated semaglutide well, with no reported gastrointestinal side effects. Blood pressure normalized, and lipid profile improved steadily.

## Discussion

This case illustrates the potential of semaglutide to reverse early diabetic nephropathy in a patient with poorly controlled T2DM and contraindication to SGLT2 inhibitor therapy. Despite the proven renal benefits of SGLT2 inhibitors, their use can be hindered by side effects such as recurrent infections and hypotension, particularly in certain subgroups [1,2]. In such scenarios, GLP-1 RAs serve as a viable and increasingly supported alternative due to their pleiotropic effects on glycemia, weight, lipids, and kidney function [3]. In our patient, semaglutide initiation resulted in significant reductions in HbA1c (from 9.8% to 6.1%) and body weight (13 kg), along with improvements in lipid profile - LDL reduced from 202 mg/dL to 80 mg/dL and triglycerides from 295 to 128 mg/dL. Most notably, renal function showed progressive improvement, with eGFR increasing from 56 to 71 mL/min and ACR decreasing by nearly 90% over the 12-month follow-up. These outcomes align with clinical trial findings. The SUSTAIN-6 trial revealed a significant reduction in new or worsening nephropathy with semaglutide, primarily due to reductions in macroalbuminuria [3]. The REWIND trial confirmed the renoprotective effects of dulaglutide across a broad spectrum of T2DM patients [7]. The FLOW trial, designed specifically to evaluate kidney outcomes, demonstrated that semaglutide slowed the progression to ESRD and attenuated eGFR decline [8]. The mechanisms behind these benefits include improved insulin sensitivity, anti-inflammatory and antioxidant effects, enhanced endothelial function, natriuresis, and blood pressure reduction [4-6]. Moreover, semaglutide can ameliorate hepatic steatosis and dyslipidemia, further improve metabolic health, and indirectly support renal recovery [11,12]. In summary, this case reinforces the clinical utility of GLP-1 RAs in managing diabetic nephropathy, particularly in patients who cannot tolerate or do not respond adequately to SGLT2 inhibitors. Semaglutide offers comprehensive metabolic and renal benefits that support its inclusion in multidisciplinary diabetes care strategies.

## Conclusions

This case highlights the significant clinical impact of semaglutide in managing diabetic nephropathy - improving urine albumin creatinine ratio and eGFR, particularly in patients unable to tolerate SGLT2 inhibitors. Over a 12-month period, the patient showed marked improvement in renal function, glycemic control, lipid profile, and weight. These outcomes underscore the potential of GLP-1 receptor agonists as effective, multidimensional agents in diabetic care. With proper monitoring and dose titration, semaglutide may offer renal and cardiovascular benefits alongside glycemic management. This case supports considering GLP-1 RAs earlier in the treatment algorithm for patients with early diabetic nephropathy.
